# Trajectory Surface Hopping for a Polarizable Embedding QM/MM Formulation

**DOI:** 10.1021/acs.jpca.2c04756

**Published:** 2022-09-15

**Authors:** Mattia Bondanza, Baptiste Demoulin, Filippo Lipparini, Mario Barbatti, Benedetta Mennucci

**Affiliations:** † Dipartimento di Chimica e Chimica Industriale, Università di Pisa, Via G. Moruzzi 13, 56124 Pisa, Italy; ‡ Aix Marseille University, CNRS, ICR, 13385 Marseille, France; § Institut Universitaire de France, 75231 Paris, France

## Abstract

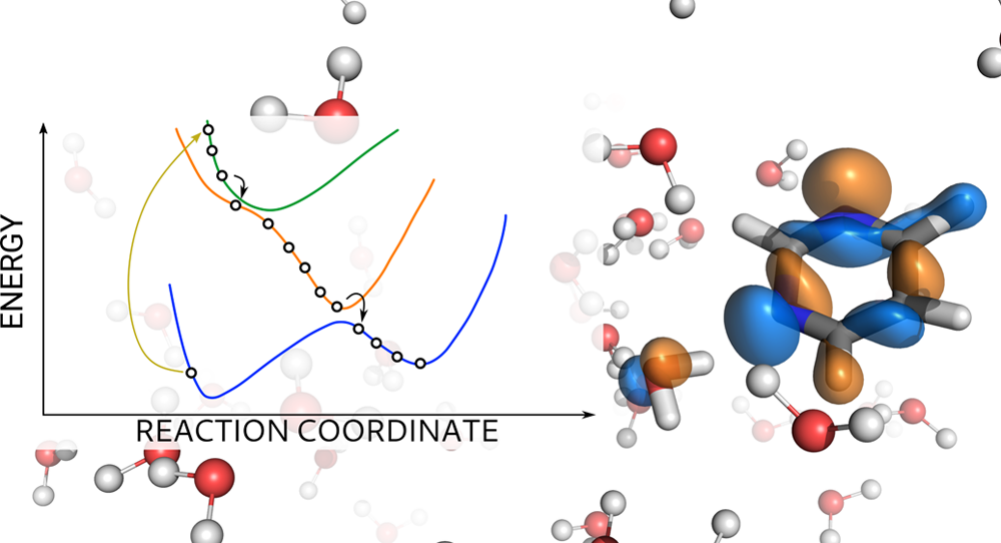

We present the implementation of trajectory surface-hopping nonadiabatic dynamics for a polarizable embedding QM/MM formulation. Time-dependent density functional theory was used at the quantum mechanical level of theory, whereas the molecular mechanics description involved the polarizable AMOEBA force field. This implementation has been obtained by integrating the surface-hopping program Newton-X NS with an interface between the Gaussian 16 and the Tinker suites of codes to calculate QM/AMOEBA energies and forces. The implementation has been tested on a photoinduced electron-driven proton-transfer reaction involving pyrimidine and a hydrogen-bonded water surrounded by a small cluster of water molecules and within a large water droplet.

## 1 Introduction

Computational photochemistry is a mature research field, but its application to complex systems remains challenging. The problem is, in fact, 2-fold: from one side, the essence of photochemistry is connected to the quantum behavior of nuclear wave packets, which is something challenging to reconstruct computationally; on the other side, the motion of such wave packets is determined by the electronic structure of the molecule itself, which can be accurately computed only with techniques having a very unfavorable scaling. Finally, both the electronic and the nuclear degrees of freedom of the system undergoing the photochemical process are coupled to the embedding environment, which can enormously enhance both the difficulty of the calculations and their computational cost.

An effective strategy to make the computational simulation feasible without losing too much accuracy is introducing a mixed quantum–classical description for modeling both dynamics and potential energy surfaces (PES). The mixed quantum–classical reformulation of dynamics allows introducing the concept of nuclear trajectories in a way that closely resembles Newton’s dynamics, thus drastically reducing the computational effort needed to simulate a wave packet propagation.^1−4^ On the other hand, the mixed quantum–classical calculation of energy, forces, and state couplings allows one to keep an accurate quantum mechanical (QM) description for the most relevant part of the system while introducing the effect of the rest through classical interactions.

A successful example of this strategy is the coupling between the trajectory surface-hopping (TSH)^5^ nonadiabatic method with a hybrid QM/MM (quantum mechanics/molecular mechanics) description.^6−9^ This approach has often been applied to solvated systems and molecular systems embedded in more complex environments such as biological macrostructures or solid matrices.^10,11^ In those applications, the usual QM/MM description adopted is an electrostatic type of embedding where the effect of the MM atoms on the QM subsystem is represented in terms of the electrostatic potential generated by the fixed atomic charges used in MM force fields to describe electrostatic interactions.^12^ In recent years, however, polarizable QM/MM formulations have rapidly diffused,^13−20^ especially for simulating light-induced processes of (multi)chromophoric systems.^21−23^

Extending polarizable models to nonadiabatic dynamics presents an inherent challenge due to the nonlinearity introduced into the system’s Hamiltonian through the dependence of the polarization degrees of freedom of the environment on the QM charge density.^22−24^ Consequently, different electronic states are eigenfunctions of different nonlinear Hamiltonians, and their orthogonality is no longer conserved. The practical realization of TSH using such a state-specific (SS) environment model is not obvious neither unique, but different approximate strategies can be envisioned, as some of the present authors have discussed in a previous article.^22^ However, if the QM method used for TSH simulation is based on a linear-response (LR) formulation, such as in time-dependent density functional theory (TD-DFT), the coupling to a polarizable model can be formulated in a much simpler way. Within this theoretical framework, in fact, the response of the polarizable environment can be recast in such a way that it does not depend on the charge density of a specific state but on transition densities. The resulting LR response, which, in the literature, has also been described as a dispersion-like interaction,^25^ makes the TD-DFT problem solvable for all of the states simultaneously exactly as for an isolated QM system.

As the LR and the SS responses describe two different physical interactions, they should simultaneously be taken into account to get a complete description of the environment effect.^25−27^ Within the TD-DFT description, the SS correction can be approximately recovered through the so-called corrected linear-response method (cLR), initially developed for continuum solvation models,^28^ but successively extended to polarizable QM/MM descriptions.^17,29^

Here, we investigate the possibilities TSH offers when combined with TD-DFT and an induced point dipole (IPD) formulation of the polarizable embedding by coupling the surface-hopping engine Newton-X^30^ with the polarizable QM/AMOEBA approach^17^ developed interfacing the Tinker^31^ and Gaussian^32^ suites of codes.

We tested this implementation on the photodynamics of pyrimidine–water clusters and on a pyrimidine molecule solvated in a water droplet. These systems are tailored for these tests because, first, we can count on previous experimental and theoretical benchmark results,^33,34^ and, second, their ultrafast dynamics and the chromophore’s small size limit the computational costs, allowing the simulation of multiple data sets.

## 2 Methods and Implementation

### 2.1 Ground and Excited States with QM/AMOEBA

In the following, we assume a Kohn–Sham (KS) density functional theory (DFT) description of the molecular system within an environment described with the AMOEBA polarizable force field.^35^ The QM/AMOEBA self-consistent polarization problem can be derived starting from the following polarization Lagrangian^24^
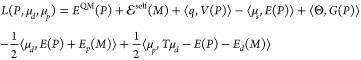
1In eq 1, the brackets denote a dot product, *P* is the density of the quantum system, *M* is the AMOEBA multipolar distribution of static charges *q*, dipoles μ_*s*_, and quadrupoles Θ_*s*_, *V*, *E*, and *G* are the electrostatic potential, field, and field gradient, whereas *T* is the dipole interaction tensor. *E*_*p*_(*M*) and *E*_*d*_(*M*) are fields produced by the multipolar distribution at the polarizable sites. They differ among themselves because of different exclusion rules used to assemble them, as from the definition of the AMOEBA force field.^35,36^ As a result, two sets of induced dipoles are generated μ_*d*_ and μ_*p*_.

By imposing the stationarity of the Lagrangian with respect to the density matrix, subject to the usual idempotency constraints, to the induced dipoles μ_*d*_ and μ_*p*_, we get an effective Kohn–Sham equation and the following equations for the induced dipoles
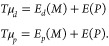
2In the effective Kohn–Sham equation, the operator contains additional terms due to the presence of the distribution *M* and a polarization term which can be recast in the following form
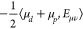
3where *E*_*μν*_ are electric field one-electron integrals. As the induced dipoles depend on the QM density matrix, the QM and polarization equations are coupled, and by solving them together, mutual polarization of the QM density and AMOEBA-induced dipoles is achieved. From a numerical point of view, this nonlinearity is not an issue for standard self-consistent field (SCF) implementations, as they already self-consistently solve a nonlinear eigenvalue problem. When a polarizable embedding is added, one has to modify the SCF algorithm by including, at each SCF iteration, the calculation of the induced dipoles so that one can add the polarization contribution to the effective Kohn–Sham matrix.^17^ Thus, polarizable embedding models are considerably more computationally demanding than electrostatic embedding QM/MM. In our implementation, the polarization linear systems in eq 2 are solved iteratively using a preconditioned conjugate gradient strategy^37^ and the fast multipole method^38^ (FMM) to compute the required matrix–vector products in a linear-scaling fashion.^39,40^

In an LR TD-DFT scheme, excitation frequencies ω are obtained by solving the generalized eigenvalue problem^41^
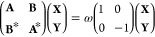
4where the **A** and **B** matrices are components of the orbital rotation Hessian and **X** and **Y** are the off-diagonal blocks of the transition density matrix. It is important to underline that solving eq 4 does not provide excited state densities. Nevertheless, they can be reconstructed by first solving a set of coupled-perturbed equations, usually called Z-vector equations.^42,43^

The effect of a polarizable environment on the description of excitation processes is far from trivial. The self-consistent polarization interaction gives rise to an additional term in the definition of the **A** and **B** matrices, thus modifying the molecular response function. In particular, both matrices are augmented by
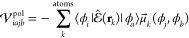
5where  is the electric field operator at **R** and  is the dipole at site *k* induced by the density element ϕ_*j*_ϕ_*b*_.^17^ At convergence, this LR environment term gives rise to an excitation energy contribution determined by the electric field generated by the transition density (**X** + **Y**) at the polarizable sites and the AMOEBA dipoles induced by the same field, namely, −⟨μ^**X**+**Y**^, *E*^**X**+**Y**^⟩. Such LR term is the instantaneous response of the AMOEBA polarizable sites to the transition density of the electronic excitation.

The same computational considerations made for the SCF apply here as well. In particular, a polarization linear system needs to be solved at each iteration of the solution of the TD-DFT equations, which is again done using our FMM-based implementation.^40,44^ From eq 5, we can see that in the LR formulation the environment’s dipoles are not relaxed on the excited state density but for each state their response to the state transition density is computed within the solution of the TD-DFT equations. This formulation makes the calculation of analytical gradients feasible using precisely the same approach used for an isolated QM system as described in detail in ref (44).

As reported in the Introduction, it is possible to recover the additional type of response due to the polarizable environment, namely, the one due to the change in the electronic charge densities of the ground and excited states (the SS effect) through the corrected LR (cLR). Within AMOEBA, the cLR correction to the excitation energy reads^17^
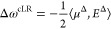
6where  is the electric field generated by the change in the electronic charge densities of the ground and excited states at the polarizable site *k* and μ^Δ^ are the induced dipoles generated by the same field.

To simultaneously take into account LR and SS responses, a simple but effective protocol has been proposed for continuum solvation models and called cLR^2^,^27^ but it can equivalently be applied to a QM/AMOEBA description. The only problem is that analytical gradients of the corrected energies have never been developed as they are cumbersome and costly to compute; for this reason, in the following, only an LR formulation will be used in the TSH dynamics. SS effects will be exclusively considered as a posteriori correction of the LR trajectories, precisely as proposed in the original cLR^2^ protocol.^27^

### 2.2 Nonadiabatic Dynamics with QM/AMOEBA

In the present work, we focus on the coupling of TSH with LR-TD-DFT performed with polarizable embedding in the IPD formulation. To implement such a methodology, we developed an interface between the TSH software Newton-X^30^ and a modified version of Tinker^31^ that can, together with a modified version of the Gaussian 16 suite of codes,^32^ compute energies and geometrical gradients of the TD-DFT/AMOEBA Hamiltonian.

**Figure 1:**
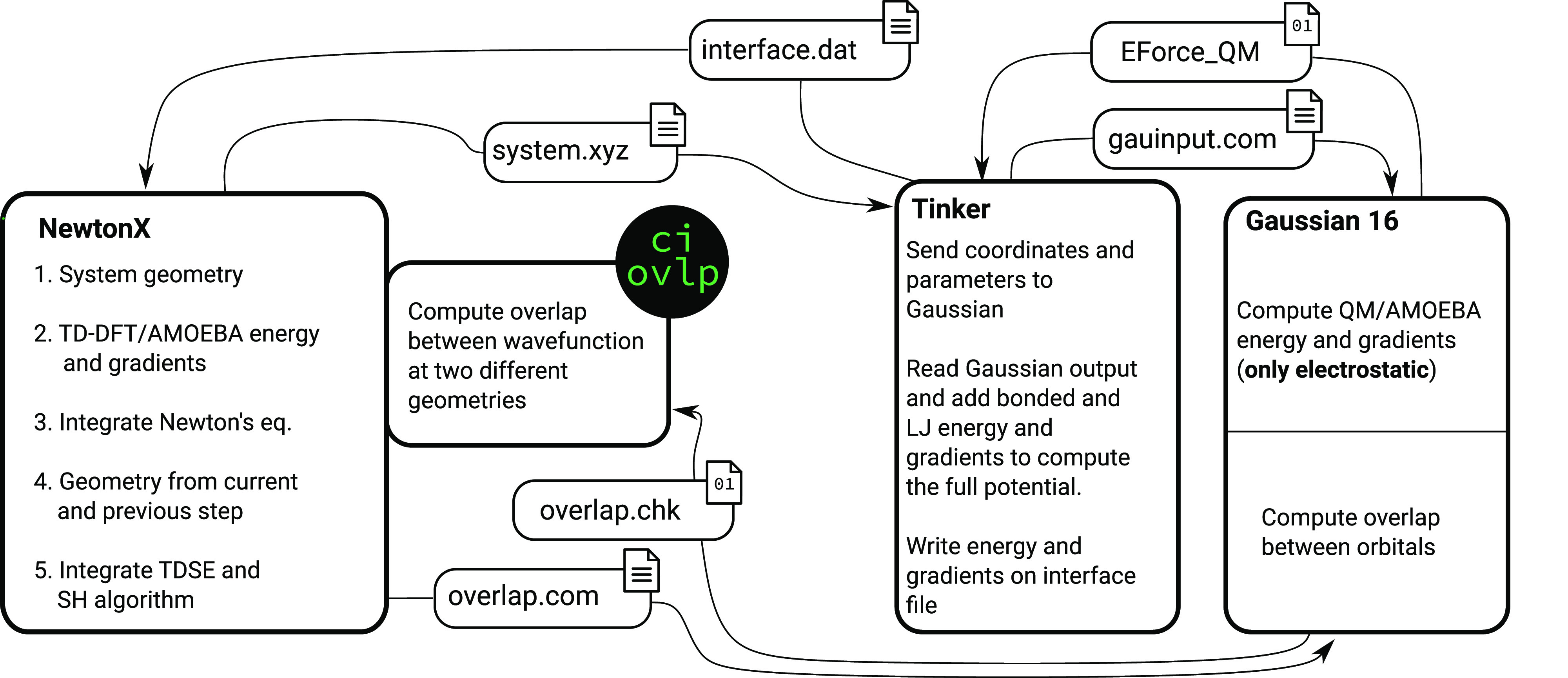
Schematic representation of Newton-X-Tinker-Gaussian implementation. Newton-X acts as a driver providing at the beginning of each step the coordinates of the system to Tinker that in turn calls Gaussian to compute energies and gradients with TD-DFT/AMOEBA PES. Then, to evaluate the transition probabilities between states, the overlap of wave functions at two subsequent time steps are computed by a program written on purpose (ci ovlp in the scheme, it is provided together with Newton-X) that uses the orbital overlap matrix computed using Gaussian.

In our implementation (Figure 1), Newton-X NS (*new series*) handles the time propagation and the surface-hopping algorithm. At each step of the dynamics, it calls the Tinker/Gaussian interface to compute the required electronic-structure quantities: (a) the potential energies, (b) the potential energy gradients, (c) the single-excitation coefficients (corresponding to the elements of the **X** + **Y** matrix), and (d) the molecular to atomic orbitals transformation matrix. The latter two are required to evaluate the overlap between Casida’s wave functions, used to estimate time-derivative nonadiabatic couplings.^45,46^

In particular, auxiliary wave functions for each electronic excited state *I* are written as^47^
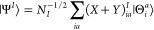
7where  is the normalization factor and  is a Slater determinant with an electron promoted from Kohn–Sham spin–orbital orbital *i* to *a*. With this wave function definition, we can compute the time-derivative nonadiabatic coupling^5,46^
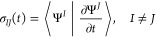
8employing the Hammes-Schiffer and Tully (HST) approach^48^
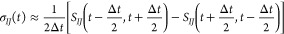
9where *S*_*IJ*_ are the overlap terms
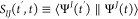
10Either adopting the determinant-derivative^49−51^ or the orbital-derivative approach,^52^ calculating these overlaps reduces to determining the atomic-orbital overlap matrix between sequential time steps. Within this formalism, the presence of a polarizable MM environment does not introduce any specificity as its effects are implicitly accounted for through the modifications induced in the wave functions.

Gaussian is used to compute ground and excited state energies and gradients considering the electrostatic and polarization part of the AMOEBA force field, the TD-DFT, and their coupling. Energies and gradients of the bonding components of the force field together with those of van der Waals nonbonding interactions are evaluated by Tinker, which adds them to the terms computed by Gaussian. To keep this framework unchanged, given the system’s geometry, we wrote an interface tool within the Tinker package which allows computing single-point energies and gradients for the LR-TD-DFT/AMOEBA potential. When such a calculation is performed, Tinker computes bonding and van der Waals nonbonding interactions for the system, creates the input for Gaussian, runs it, and finally collects all of the results together, printing energies and forces on a formatted interface file.

All of the implementations described up to now are self-contained in a Fortran module and a Perl script used to interface Newton-X NS and Tinker/Gaussian, providing the data needed to the core code in the correct format (and units). The interface between Newton-X and Tinker/Gaussian is handled with two formatted files (system.xyz and interface.dat, Figure 1) containing the coordinates of the system and its energy and gradients, respectively. Even if formatted files are clearly less efficient than binary ones or direct in-memory communication, we decided to adopt this solution as, at the current stage, it does represent a minor overhead with respect to the cost of the on-the-fly evaluation of PESs and gradients while allowing a much easier debug and development. Since the TSH core code was not ready to deal with QM/MM calculations, some minor modifications have also been performed. In particular, manipulation of the system’s kinetic energy is needed to handle frustrated hoppings, velocity rescaling after hopping, and decoherence corrections.^53^ When a QM/MM potential is used, it is more sound to exclude atoms from the environment and to only use the kinetic energy of the QM part. Otherwise, the amount of kinetic energy available for these effects becomes anomalously large, leading to size-extensivity errors when the direction of the nonadiabatic coupling vector is not explicitly known.^54^

## 3 Test Application

As a test case of the TSH with TD-DFT/AMOEBA implementation, we focused on a pyrimidine (Pm) water cluster.

**Figure 2:**
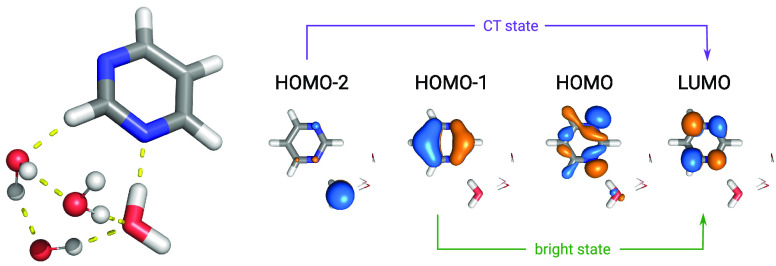
Graphical representation of the clusters Pm(H_2_O)_4_ (left); hydrogen bonding is represented with dashed yellow lines, molecules in the MM region are represented with balls and sticks, while those in the QM region are represented as licorice. Relevant transitions for the EDPT process represented as single excitations between molecular orbitals at the equilibrium geometry of Pm(H_2_O)_4_. MOs are computed with QM/AMOEBA Hamiltonian and plotted as isosurfaces at +0.01 and −0.01 in orange and blue, respectively.

The photoinduced process in nitrogen aromatic heterocycles interacting with water molecules has been accurately investigated, combining laser spectroscopy, mass spectrometry, and QM calculations.^33,34,55−58^ These investigations show that the mechanism involves a photoinduced electron-driven proton-transfer (EDPT) reaction,^59^ representing a subcase of the proton-coupled electron-transfer (PCET) mechanism,^60^ namely, after photoexcitation of the aromatic heterocycle, an electron is transferred from the lone pair of an H-bonded water to its π* orbital (see Figure 2). The resulting charge-transfer (CT) state is quickly neutralized by the displacement of the H-bonding proton, forming a new bond with the aromatic nitrogen. After the EDPT, the system is still excited but it quickly relaxes to the ground state (GS), which is a biradical at this geometry.

Huang et al.^33^ also showed that a TSH algorithm combined with the algebraic diagrammatic construction to the second-order [ADC(2)], a single-reference method like TD-DFT, could adequately describe the excited-state process until the system relaxed to the ground state, which has a diradical character.

Here, we followed a similar computational protocol for the Pm(H_2_O)_4_ system using TD-DFT as the QM method for Pm and the water molecule H bonded to a Pm nitrogen atom and AMOEBA for the three “spectator” water molecules (see Figure 2).

### 3.1 Computational Details

Due to the importance of accurately describing CT states, we used the long-range-corrected functional CAM-B3LYP,^61^ which should provide a balanced representation of the different electronic states involved in the process. The calculations were done with the 6-31+G(d) basis set. To further test the robustness of the results, we selected a sample trajectory and recalculated the energy of each electronic state by enlarging the basis set on the hydrogen atoms (e.g., switching to 6-31+G(d,p) or even 6-31++G(d,p)). The obtained results show very small (<0.05 eV) changes in the absolute values of the energies of the different electronic states and no changes in their relative order.

All TSH simulations were performed applying the same protocol. We started from the optimized structure of the cluster; then, we computed the Hessian with respect to the nuclear coordinates. Geometry optimizations for QM/AMOEBA and QM/TIP3P were performed with the program minimize.x from the Tinker package using a threshold of 0.1 kcal mol^–1^ Å^–1^ on the gradients’ root-mean-square. Since analytical second derivatives are not implemented in our software for QM/AMOEBA Hamiltonians, we used numerical differences of analytical gradients with a modified version of vibrate.x program from the Tinker package. Ground state equilibrium geometries and Hessians were used to sample 512 structures from a harmonic-oscillator Wigner distribution^62^ of the clusters using Newton-X.^30,63^ On each structure, we computed the lowest five excitation energies at the same level of theory. Finally, we selected initial conditions from each state according to a stochastic algorithm based on the state’s oscillator strength within a 0.5 eV energy width around the equilibrium excitation energy of S_4_.

Preparation of the initial conditions for the pyrimidine–water droplet was performed differently: we took 128 configurations extracted from the Wigner distribution of Pm(H_2_O)_4_ and using PACKMOL^64^ added 1148 water molecules inside a sphere of 20 Å radius around the original cluster. Then, we performed a short Born–Oppenheimer dynamics simulation in the ground state to equilibrate the solvent. This simulation kept the QM region (the Pm molecule and the water H bound to the nitrogen atom) rigid, while all of the classical waters were free to move. The Berendsen thermostat was applied with a τ constant of 0.05 ps^–1^ and a reference temperature of 300 K. A harmonic wall (as implemented in Tinker) kept the molecules within 20 Å from the system’s center. The dynamics was performed with a time step of 0.5 fs for a total length of 1 ps. We noticed that the temperature was stabilized after about 500 fs. Thus, for each trajectory, we extracted two configurations spaced by 250 fs from the second part of the equilibration. On those structures, we computed excitation energies and performed the sampling of the initial conditions, as described above for the Pm(H_2_O)_4_ cluster.

Each initial condition was used for a single trajectory. TSH was run with the decoherence-corrected^53^ fewest switches surface-hopping^5^ (DC-FSSH) approach. Time-derivative nonadiabatic couplings were calculated with the orbital-derivative approach^52^ as described in the Methods and Implementation. Each trajectory was propagated as a microcanonical ensemble for a maximum of 100 fs with a time step of 0.5 fs for the Newton’s equation integration; the time step for integration of the time-dependent Schrödinger equation was 0.025 fs with electronic properties interpolated between classical steps. The decoherence correction by Granucci and Persico^53^ was applied with a parameter of 0.1 Hartree. After a frustrated hopping, the velocity was kept in the original direction. After a hopping, the velocity was rescaled in the momentum direction. As mentioned in the Methods and Implementation, only QM kinetic energies were considered for decoherence, frustrated hoppings, and velocity rescaling.

Due to limitations of TD-DFT to describe the crossing region between the ground and the first excited state,^46^ only hoppings between excited states were evaluated. Whenever the S_1_/S_0_ energy gap dropped below 0.2 eV, the trajectory was terminated and the corresponding time was taken as the time for the internal conversion to the ground state. Trajectories were considered valid only if they were longer than 5 fs. They were terminated if they underwent a total energy drift superior to 0.5 eV.

The trajectories were analyzed using a Python code based on NumPy,^65,66^ SciPy,^67^ and MDAnalysis^68^ libraries. Data visualization was performed using Matplotlib,^69^ while molecular visualization was performed using PyMOL. Automatic analysis of the TD transition density matrices was performed with TheoDORE.^70^

### 3.2 Results

We ran three different sets of TSH simulations to establish a robust data set to test the implementation of TD-DFT/AMOEBA. The first reference set focused on Pm(H_2_O)_4_ with pyrimidine and one water molecule in the QM (TD-DFT) and three water molecules in the MM (AMOEBA) regions (Figure 2). The second set adopted the same QM and MM regions but with regular electrostatic embedding using the TIP3P force field. The third set stripped the spectator water molecules and simulated only Pm(H_2_O)_1_ with TD-DFT.

**Figure 3:**
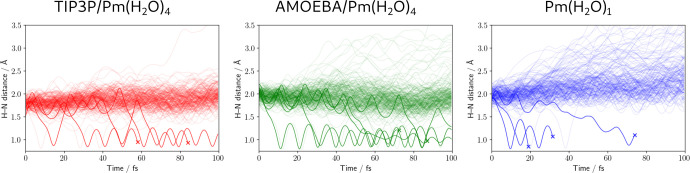
H–N distance computed along TSH trajectories of QM/TIP3P (left), QM/AMOEBA (center), and Pm(H_2_O)_1_ (right). Lines corresponding to EDPT events are highlighted; final cross indicates that the trajectory is terminated before 100 fs.

As a first analysis, we compare the EDPT probabilities in the three trajectory sets. The results are shown in Figure 3, where we report the Pm(N)–H(water) distance computed along TSH trajectories of QM/AMOEBA, QM/TIP3P, and Pm(H_2_O)_1_. As it can be seen, for all three sets of trajectories, the H transfer occurs in the first 100 fs of the excited state radiationless decay. When we compare the EDPT events we see only slight differences among the three sets: 5 in 222 trajectories (2.2%) for QM/AMOEBA, 3 in 220 (1.4%) for QM/TIP3P, and 3 in 190 for Pm(H_2_O)_1_ (1.6%). For a 95% confidence interval, the margin of error is ±2% in the three cases, meaning these results agree with each other within the statistical uncertainty.

Figure 3 also shows that in Pm(H_2_O)_1_ many trajectories lead to a breaking of the H bond (34 ± 7%), while this behavior is largely reduced in QM/AMOEBA (7 ± 3% of trajectories with N–H distance reaching over 2.5 Å) and almost canceled in QM/TIP3P (1 ± 1%). The difference between Pm(H_2_O)_1_ and Pm(H_2_O)_4_ reflects the chemical intuition that an H-bonding network contributes to the system’s stability. On the other hand, the larger propensity of the QM/AMOEBA cluster to break apart the N(Pm)–H(water) hydrogen bond with respect to QM/TIP3P can be interpreted as the result of a *softer* nature of the polarizable AMOEBA force field with respect to TIP3P.

We also performed a fourth set of TSH trajectories using a full-QM Pm(H_2_O)_4_ cluster. In this case, the EDPT probability is significantly increased, with 26 out of 167 trajectories (16 ± 6%) showing the H transfer (see Figure S1 of the SI). We recall that in the previous ADC(2) simulations of the same cluster^33^ the EDPT event was seen with a probability of 6%. Analyzing a reactive trajectory and evaluating the character of the different excited states in time (see Figure S2 of the SI), we have seen low-lying charge-transfer states involving orbitals delocalized over all water molecules. As a result, the H-transfer mechanism significantly changes as the EDPT is no longer localized on the water directly H bonded to the Pm nitrogen. Because of that, the comparison with QM/MM descriptions is no longer meaningful and the full-QM Pm(H_2_O)_4_ model will no longer be considered.

**Figure 4:**
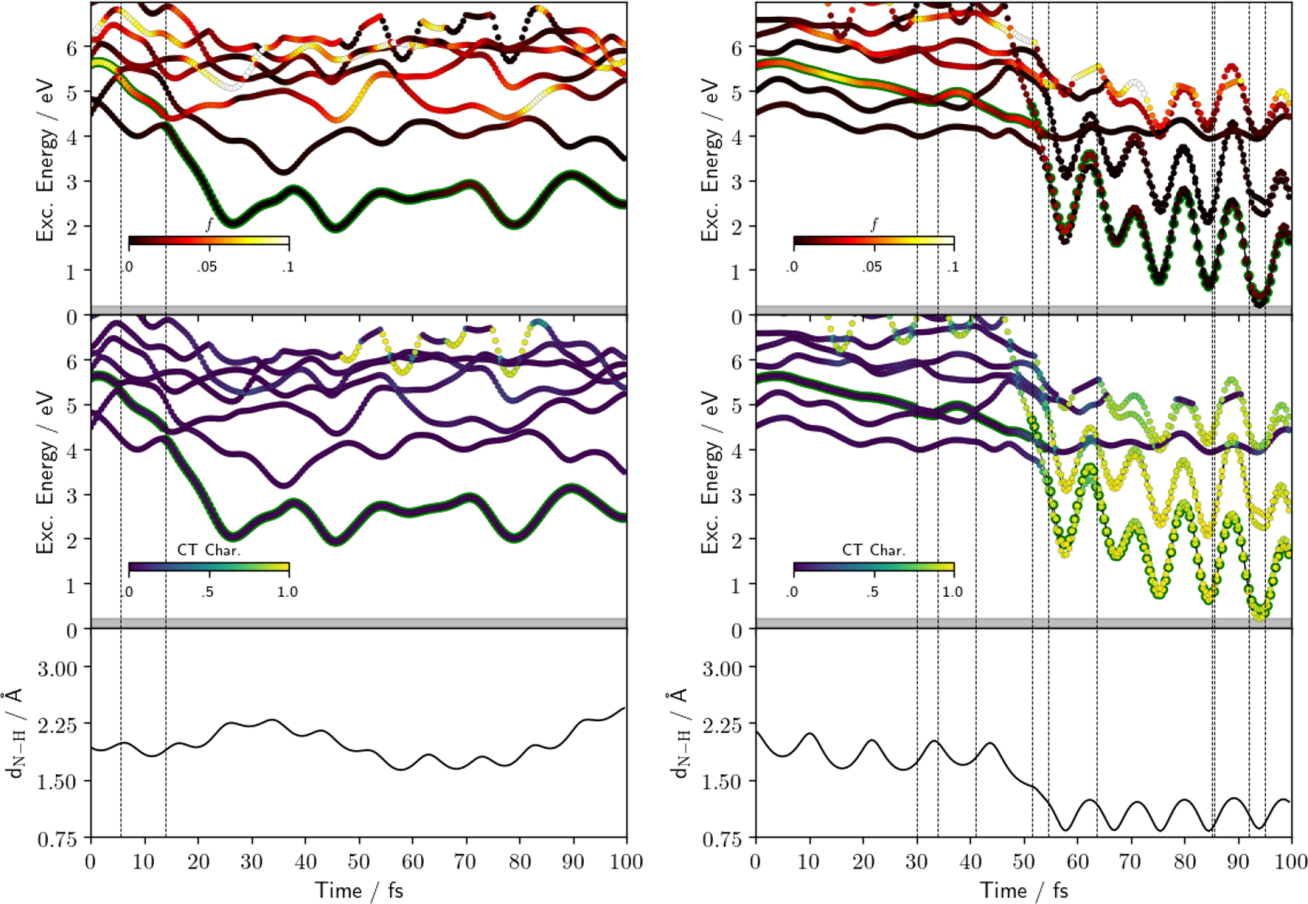
Comparison of a nonreactive (left) and a reactive QM/AMOEBA trajectory (right). (Top) Excitation energies of the low-lying states colored in terms of the intensity of their oscillator strength (see color bar inside the picture); state with green edges is the current state of TSH, vertical lines indicate the hopping events, and gray shadow is the region where the simulation is stopped and considered as relaxed on the GS. (Middle) Same as the top one but the color maps the  character of the states. (Bottom) Distance of Pm N atom from the hydrogen atom of the initially H-bonded water molecule.

To have a more direct analysis of the process, we compared two representative examples of reactive and nonreactive QM/AMOEBA trajectories. For each frame of the two trajectories, we computed the transition density matrices for the lowest lying states and analyzed them with TheoDORE.^70^ In Figure 4, we show the oscillator strengths (*f*) and CT character of the different electronic states along the two trajectories; the N–H distance is also reported for reference. To compute the CT character of a state from the transition density matrices, the system has to be partitioned into molecular fragments; since the molecular entities do change upon EDPT, we adopted a *dynamic* definition of the molecular fragments based on the N–H bond distance. Using such a definition, during the first part of a reactive trajectory, the fragments are Pm and water, while they change into PmH and OH after EDPT. These graphs show a straightforward behavior for the nonreactive trajectory: the bright ππ* state populated by light absorption quickly relaxes to a dark state (nπ* in character), which evolves in time, substantially lowering its energy from the initial ∼5.5 eV to less than 3 eV. During the dynamics, we observe the presence of some states with a significant CT character but always well separated (much more than 1 eV) from the current state.

The reactive trajectory still starts on a bright state in a situation that does not seem very different from the previous one. However, the dynamics now follows a different path: for the first ∼50 fs, the bright state remains populated until it jumps to a low-lying (dark) CT state. When the CT state is initially populated, the N–H distance is still above 1.5 Å, indicating that the electron transfer (populating the CT state) causes the subsequent proton transfer, as previously found in the ADC(2) simulations.^33^ To better interpret the graphs reported in Figure 4, we recall that the definition of the CT character of each excited state depends on the reference ground state.^70^ Since we are using a closed-shell representation, after EDPT, the GS is described as an ion pair (PmH^+^···OH^–^), and as a consequence, the diradical excited states (PmH^•^ ··· OH^•^) present a high CT character with respect to this reference.

**Figure 5:**
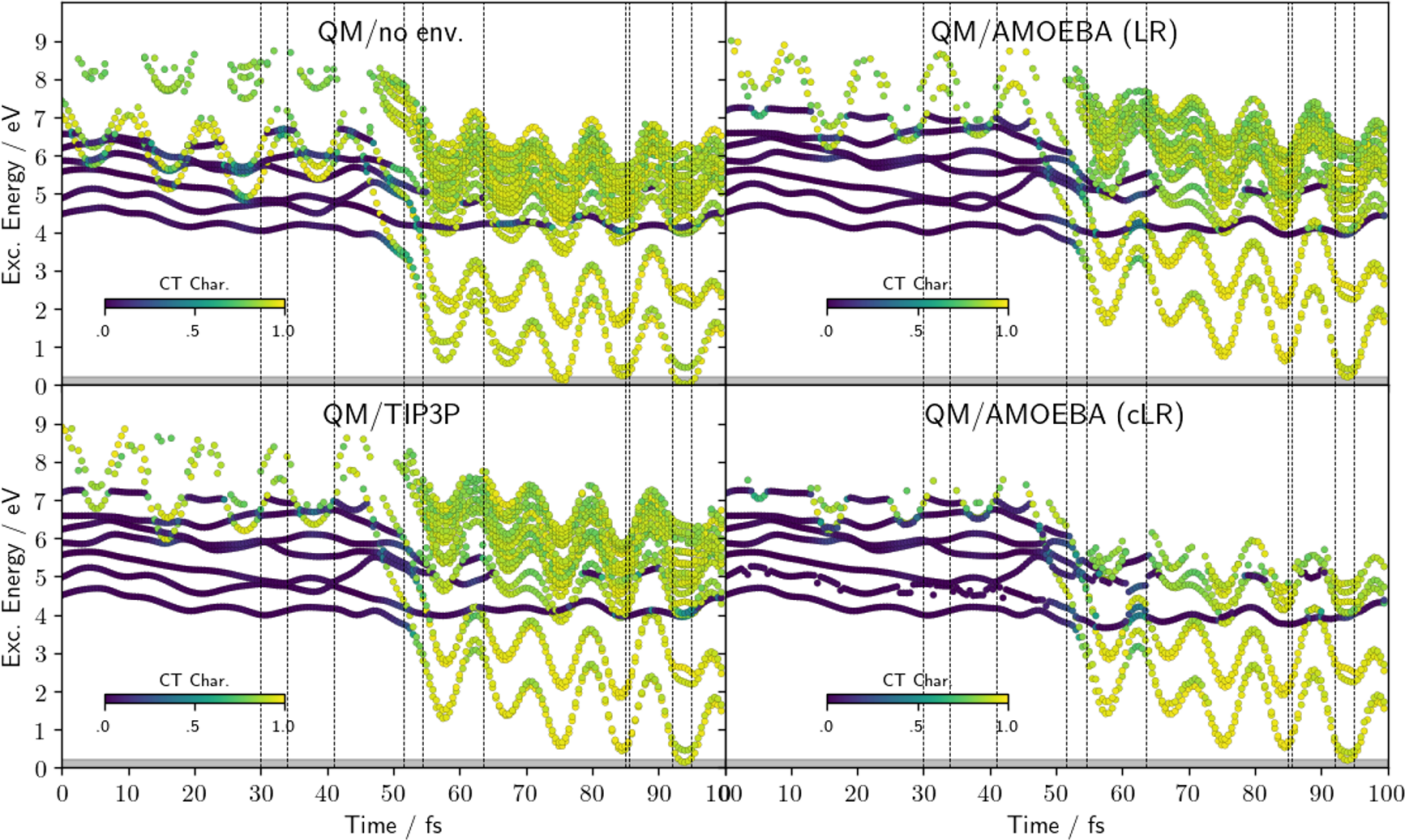
Energies and CT character of the lowest excited states computed along the QM/AMOEBA reactive trajectory shown in Figure 4 with different representations of environment water molecules: omitted (upper left), LR QM/AMOEBA (upper right), QM/TIP3P (bottom left), and cLR^2^ QM/AMOEBA for each state (bottom right). In the latter case, only the lowest 10 excited state have been analyzed.

We further investigated the energy and the character of the states in time. To do so, we considered the same QM/AMOEBA reactive trajectory already analyzed before (see Figure 4), and we recomputed the excited states using different models for the spectator waters, namely, neglecting them (no env), using TIP3P, and correcting QM/AMOEBA for a state-specific effect using the cLR^2^ approximation commented in the Methods and Implementation section. The graphs reported in Figure 5 show these results in comparison to the original linear-response QM/AMOEBA (LR) for reference.

The presence of the additional water molecules destabilizes the CT states, increasing their energies with respect to the model with no environment. Such an effect, slightly more marked for QM/AMOEBA than for QM/TIP3P, can be explained in terms of stabilization of the lone pair orbital when in the presence of the additional water molecules. The introduction of the state-specific correction (cLR^2^) inverts this behavior, stabilizing the CT states while leaving the other states unperturbed. The global effect, however, is small due to the small number of polarizable water molecules.

**Figure 6:**
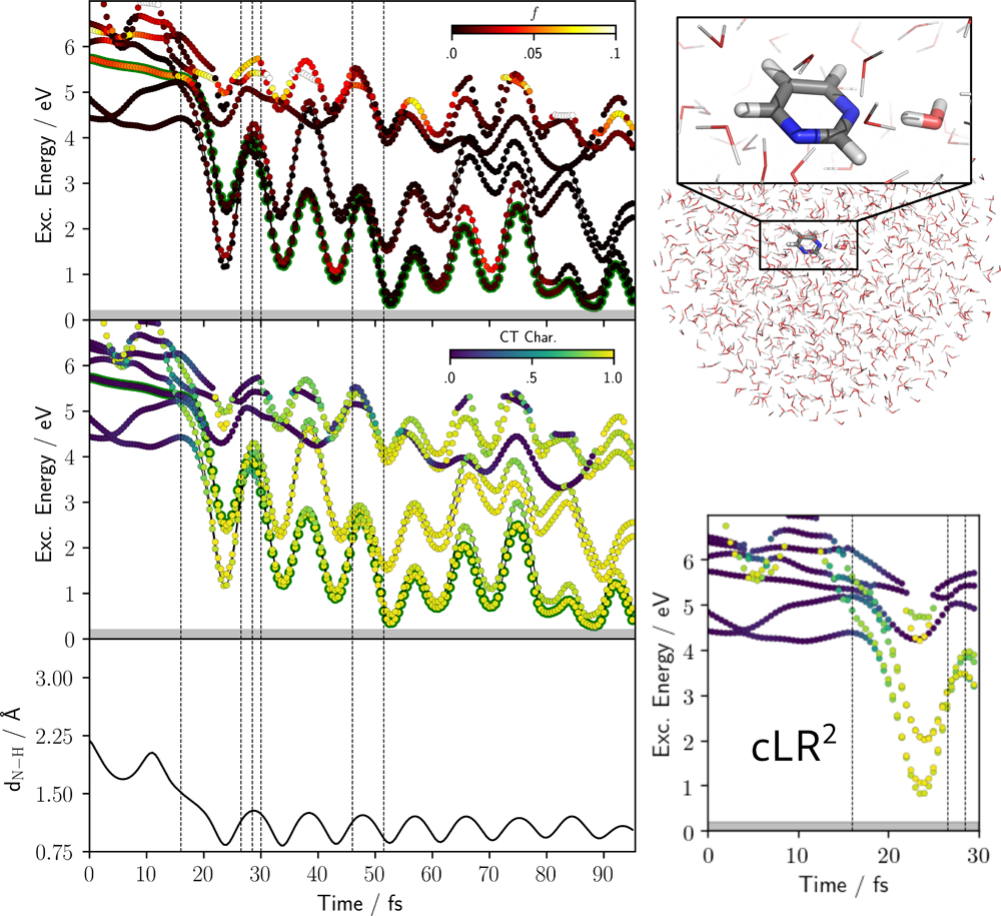
Pyrimidine water droplet (top right). At the left, analysis of a reactive trajectory for the droplet. Format is the same as that adopted in Figure 4. Bottom right shows the CT character of the first 30 fs of the same trajectory but considering cLR^2^ AMOEBA.

Following these findings, we decided to further investigate how state-specific effects could impact the description in a large (bulk-like) environment. To check this point, we ran another set of TSH trajectories for Pm(H_2_O)_1_ within a 20 Å droplet of AMOEBA water molecules (Figure 6). We found that the EDPT probability for the droplet remains close to that found for Pm(H_2_O)_4_ (2 reactive out of 81 trajectories, or 2.5 ± 3%). By analyzing a reactive trajectory, we saw that the behavior also closely resembles that already discussed for Pm(H_2_O)_4_ with a transition from a bright to a dark CT state, finally leading to the H transfer. However, when we calculated the QM/AMOEBA-cLR^2^ energies of the low-lying states using the frames from the first 30 fs of the QM/AMOEBA droplet trajectory, we found that the CT states are stabilized by a much larger amount than what was observed for Pm(H_2_O)_4_ (about 0.4 eV). One would expect that this stabilization had a not negligible effect on the EDPT probability. Unfortunately, the lack of a state-specific formulation of TSH due to the difficulties of such an implementation discussed in the Methods and Implementation section prevents a quantitative estimate of the final effect on the EDPT yield.

## 4 Conclusions

We reported the implementation of trajectory surface-hopping nonadiabatic dynamics performed with a QM method (TD-DFT) coupled to an atomistic polarizable embedding (AMOEBA). This implementation has been done by interfacing a development version, soon to be released, of the surface-hopping program Newton-X NS with the polarizable QM/AMOEBA approach from an interface between the Gaussian 16 and the Tinker suites of codes. Our NX/TINKER/G16 interface will be released under the GPL3 license, freely available to anyone with access to Gaussian 16 and the Tinker version as modified in this work. We tested our approach on photoexcited pyrimidine–water clusters, a challenging system because of the relevant role played by CT states. Our analysis led us to conclude that the performance of AMOEBA and TIP3P water models in this kind of problem is very similar if AMOEBA is treated in a linear-response framework. Instead, significant differences in the charge-transfer states’ energies are obtained when state-specific corrections are introduced in the QM/AMOEBA description. These findings push for a reliable and efficient implementation of analytical gradients of state-specific formulations of polarizable QM/MM approaches.

## Notes

All of the raw data supporting this study’s findings (complete trajectories and transition density matrices analysis) are available on figshare (10.6084/m9.figshare.20236674).

## Supporting Information Available

Results for the set of TSH trajectories calculated using a full-QM Pm(H_2_O)_4_ cluster (PDF)
