# Supplementary material for: Trajectory Surface Hopping for a Polarizable Embedding QM/MM Formulation
Source: J Phys Chem A. 2022 Sep 15;126(38):6780–9. doi: 10.1021/acs.jpca.2c04756 (PMC9527758; doi:10.1021/acs.jpca.2c04756)
Supplement: Supplementary file 1 — jp2c04756_si_001.pdf [file jp2c04756_si_001.pdf]

Supporting Information
Trajectory Surface Hopping for a Polarizable Embedding QM/MM

Mattia Bondanza,† Baptiste Demoulin,‡ Filippo Lipparini,† Mario Barbatti,*,‡,¶ and Benedetta Mennucci*,†

†*Dipartimento di Chimica e Chimica Industriale, Università di Pisa, Via G. Moruzzi 13, 56124 Pisa, Italy*

‡*Aix Marseille University, CNRS, ICR, Marseille, France*

¶*Institut Universitaire de France, 75231 Paris, France*

E-mail: mario.barbatti@univ-amu.fr; benedetta.mennucci@unipi.it

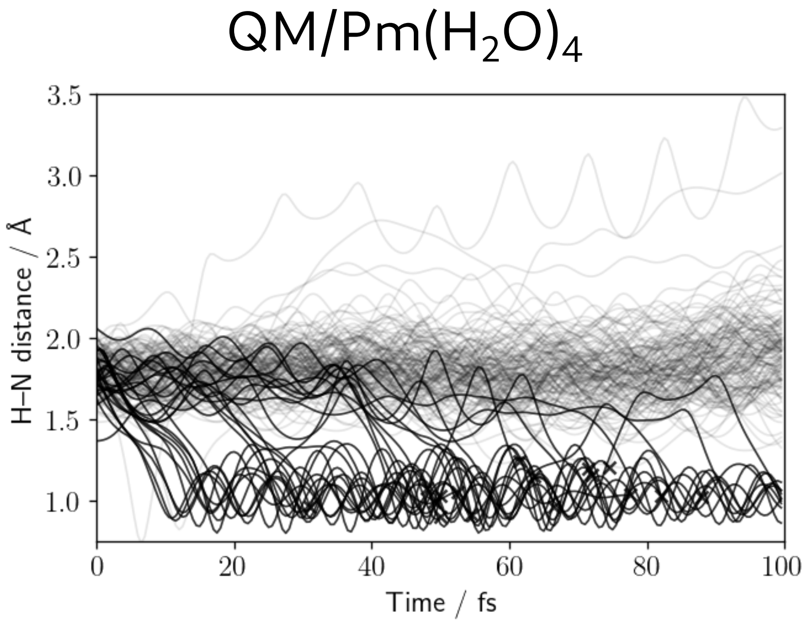

Figure S1: H-N distance computed along TSH trajectories of full-QM $Pm(H_2O)_4$. Lines corresponding to EDPT events are highlighted; the final cross indicates that the trajectory is terminated before 100 fs

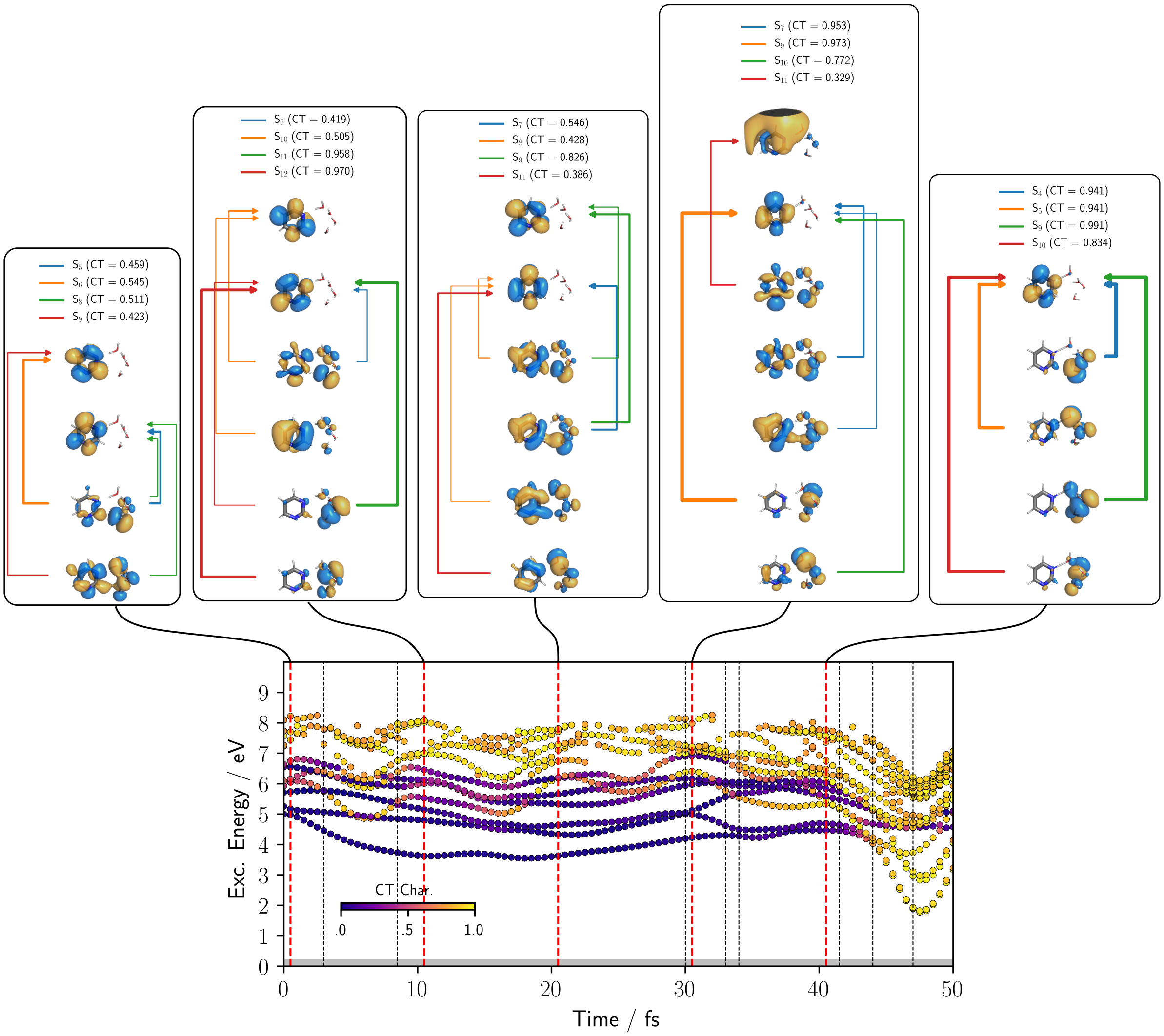

Figure S2: Decomposition of four lowest CT states (states are considered as CT if the CT character is above .75) in terms of transitions between molecular orbitals on some snapshots of the reactive trajectory shown in Figure S1. MOs are represented as isosurfaces at +/- 0.02, transitions are represented with arrows having a width proportional to the weight of the transition itself.
